# 

**Published:** 1922-09

**Authors:** 


SEPTEMBER, 1922.

*Vol. XXXIX.* *No.* **146.**

THE

### BRISTOL MEDICO-CHIRURGICAL JOURNAL

*PUBLISHED UNDER THE AUSPICES OF*

*THE BRISTOL MEDICO-CHIRURGICAL SOCIETY.*

*Editor:* P. WATSON-WILLIAMS, M.D.,

WITH WHOM ARE ASSOCIATED

JAMES SWAIN, C.B., C.B.E., M.S., M.D.,

J. LACY FIRTH, M.S., M.D., CAREY COOMBS, M.D.

J. A. NIXON, C.M.G., M.D., *Assistant Editor.*

*Editorial Secretary:* A. L. FLEMMING, M.B., Ch.B.

BRISTOL: J. W. ARROWSMITH LTD.

London: Simpkin, Marshall, Hamilton, Kent & Co. Ltd.

*Price Three Shillings net.*

